# Platinum-Sensitive Recurrence in Ovarian Cancer: The Role of Tumor Microenvironment

**DOI:** 10.3389/fonc.2013.00251

**Published:** 2013-09-24

**Authors:** Jeremy Chien, Rui Kuang, Charles Landen, Viji Shridhar

**Affiliations:** ^1^Department of Cancer Biology, University of Kansas Medical Center, Kansas City, KS, USA; ^2^Department of Computer Science and Engineering, University of Minnesota, Minneapolis, MN, USA; ^3^Department of Obstetrics and Gynecology, University of Alabama at Birmingham, Birmingham, AL, USA; ^4^Division of Experimental Pathology, Mayo Clinic, Rochester, MN, USA

**Keywords:** ovarian cancer, extracellular matrix, platinum-sensitive recurrence, platinum resistance, cancer stem cell

## Abstract

Despite several advances in the understanding of ovarian cancer pathobiology, in terms of driver genetic alterations in high-grade serous cancer, histologic heterogeneity of epithelial ovarian cancer, cell-of-origin for ovarian cancer, the survival rate from ovarian cancer is disappointingly low when compared to that of breast or prostate cancer. One of the factors contributing to the poor survival rate from ovarian cancer is the development of chemotherapy resistance following several rounds of chemotherapy. Although unicellular drug resistance mechanisms contribute to chemotherapy resistance, tumor microenvironment and the extracellular matrix (ECM), in particular, is emerging as a significant determinant of a tumor’s response to chemotherapy. In this review, we discuss the potential role of the tumor microenvironment in ovarian cancer recurrence and resistance to chemotherapy. Finally, we propose an alternative view of platinum-sensitive recurrence to describe a potential role of the ECM in the process.

The majority of patients with advanced ovarian cancer develop recurrent disease within 3 years (1, 2) and die within 5 years because relapsed disease is almost always incurable (3). Although initial recurrences are frequently platinum-sensitive, patients eventually develop resistance to platinum-based chemotherapy (3). Accordingly, resistance to chemotherapy, whether intrinsic (primary) or acquired (secondary) resistance, is a major problem in the treatment of ovarian cancer and the main contributing factor in cancer-associated mortality.

The initial response to platinum-based chemotherapy in ovarian cancer can be broadly classified into three groups: platinum-refractory, platinum-resistant, and platinum-responsive. These classifications are based mainly on clinical evidence and useful in the clinical management of ovarian cancer. Among them, the platinum-refractory group is perhaps the easiest to conceptualize because these patients do not respond to platinum-based therapy and show progression during the course of the therapy. On the other hand, platinum resistance is defined by less than 6 months of remission following chemotherapy (3). Clinically, these patients will show initial response to chemotherapy but experience relapse within 6 months of the last round of chemotherapy, a time course often described as platinum-free interval or treatment-free interval. Treatment-free interval less than 6 months is often used as a clinical cutoff to define platinum-resistant disease because of empirical evidence (4). For patients who initially respond to platinum-based therapy, there is a spectrum of response that lasts from a little over 6 months to several years.

Although several genomic studies have been conducted to identify the underlying genetic basis of this tumor behavior in response to chemotherapy, major mechanisms or biological pathways that contribute to differential response to chemotherapy are not fully understood. It is generally accepted that multiple molecular mechanisms contribute to chemotherapy resistance and that a single mechanism is unlikely to account for tumor response to chemotherapy.

Recent review by Galluzzi et al. provides an excellent conceptual view of tumor intrinsic mechanisms associated with cisplatin resistance (5). Alterations in pre-targets (associated with drug metabolism and transport before it reaches to its intracellular targets), on-targets (associated with DNA damage signaling and repair), post-targets (associated with apoptosis and survival signaling), and off-targets (components not directly affected by cisplatin but counteract the lethal effect of cisplatin) are associated with cisplatin resistance (5). Although cisplatin-resistant mechanisms are well studied and reviewed, molecular mechanisms associated with platinum-sensitive recurrence is not well understood.

An interesting aspect of ovarian cancer is that the majority of patients who relapse long after chemotherapy can be rechallenged with the same chemotherapy (4). These patients are described as having platinum-sensitive recurrence. Therefore, the traditional view of intratumor heterogeneity and the clonal selection of resistant cancer cells by chemotherapy does not fit well with the clinical evidence because the selection of resistant cells from heterogeneous tumor cell population following chemotherapy would have resulted in platinum-resistant recurrence and not platinum-sensitive recurrence. Platinum-sensitive recurrent ovarian cancer is a subject of numerous research and clinical studies because the majority of ovarian cancer patients fall into this category (2, 6). From a research point of view, platinum-sensitive recurrence is an enigma. In a traditional viewpoint, platinum-resistant or -refractory ovarian cancer can be explained by a simple model in which intrinsically resistant tumor cells from heterogeneous tumor population were selected for by chemotherapy resulting in emergence of chemotherapy-resistant or -refractory tumors (7, 8) (Figures 1A,B). It is difficult to apply this simplistic model to platinum-sensitive recurrent disease because not all tumor cells that persist through initial rounds of chemotherapy become resistant to chemotherapy. In fact, provided that patients experience long remission prior to relapse, these patients will likely respond to platinum-based chemotherapy again.

**Figure 1:**
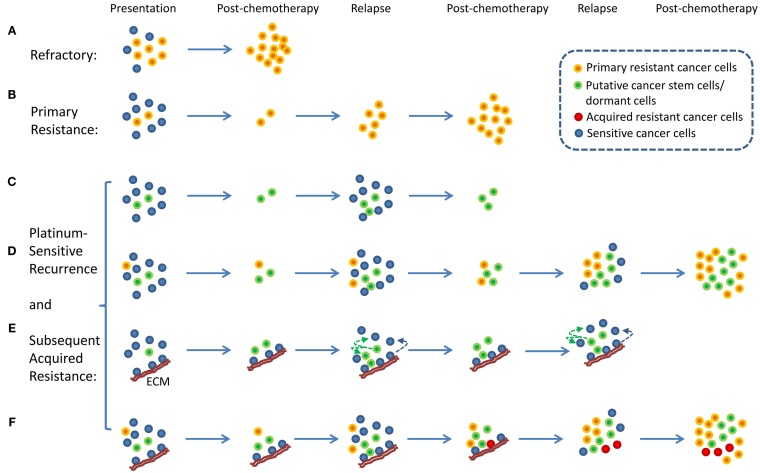
**Proposed models of platinum-resistant and platinum-sensitive recurrence**. **(A)** Platinum-refractory tumor. In this model, tumor consists mainly of chemotherapy-resistant cells. Upon treatment, these resistant cells continue to expand thereby resulting in progression during the treatment. **(B)** Platinum resistance. In this model, tumor consists of a large proportion of chemotherapy-sensitive and a small proportion of chemotherapy-resistant tumor cells. Upon treatment, chemotherapy-sensitive cells are eliminated, resulting in measurable clinical response. However, resistant tumor cells repopulate the tumor, and recurrence is observed within 6 months from the last chemotherapy. **(C)** A model of platinum-sensitive recurrence. In this model, quiescence “cancer stem cells” persist following chemotherapy, and repopulate the tumor after chemotherapy resulting in recurrence. Because repopulated tumor is derived from “cancer stem cells,” these tumor cells retain original phenotype in terms of their response to chemotherapy. Therefore, these tumors are expected to be sensitive to chemotherapy, thereby resulting in platinum-sensitive recurrence. **(D)** A model of acquired platinum resistance. In this model, original tumor is composed of heterogeneous population of tumor cells, consisting of a small population of chemotherapy-resistant tumor cells (orange colored cells), putative “cancer stem cells” (green colored cells), and chemotherapy-sensitive tumor cells (blue colored cells). Upon treatment, residual cells are composed of chemotherapy-resistant cells and cancer stem cells. Based on the cancer stem cell models, putative cancer stem cells are capable of re-initiating the tumor. Due to the high replicative potential of transit-amplifying cells that are derived from cancer stem cells, these cells may contribute the bulk of recurrent tumor. It is important to note that chemotherapy-resistant tumor cells (orange colored cells) will continue to expand and contribute to recurrence. However, due to the population expansion dynamics, these resistant cells may constitute a small proportion of tumor bulk. However, with subsequent rounds of chemotherapy, resistant tumor cell population will continue to expand to a point where they become the majority of tumor bulk. At this point, tumor will not display clinically measurable response, leading to clinical classification of acquired platinum resistance. **(E)** Alternative view of platinum-sensitive recurrence. In this model, tumor bulk is composed of heterogeneous population of tumor cells. Some tumor cells are associated with specific components of extracellular matrix (ECM), and this interact protect the cells from chemotherapy. These cells persist along with putative cancer stem cells, and both contribute to platinum-sensitive recurrence. It is important to note that this model posits that some tumor cells that grow out will lose their contact with particular components of the ECM, and they will become sensitive to chemotherapy. **(F)** Unified model of acquired resistance. In this model, the original tumor is composed of heterogeneous population of tumor cells consisting of cancer stem cells, quiescent or dormant tumor cells, chemotherapy-resistant cells, and tumor cells that are or are not associated with particular components of the ECM. Following chemotherapy, four types of tumor cells are posited to persist: chemotherapy-resistant tumor cells, cancer stem cells, quiescent or dormant tumor cells, and tumor cells associated with specific components of the ECM. All four cell types contribute to recurrent disease. After several rounds of chemotherapy, the model also posits the emergence of novel clones (red colored cells, derived from cells associated with the matrix, from dormant tumor cells, or from cancer stem cells) that acquired additional genetic alterations that allow *de novo* resistance to chemotherapy. All these cells may contribute to acquired resistance.

## Cancer Stem Cells as a Mechanism of Platinum-Sensitive Recurrence

With the emergence of cancer stem cell hypothesis, platinum-sensitive recurrence could be explained by putative cancer stem cells. Agarwal and Kaye proposed that in patients with platinum-sensitive recurrence, heterogeneous chemo-naïve tumor may contain clonal population of chemotherapy-sensitive tumor cells, quiescent, or dormant tumor cells that are resistant to chemotherapy, and chemotherapy-resistant tumor cells (Figures 1C,D). The last two groups of tumor cells may constitute a small proportion in the tumor. Therefore, upon treatment, the bulk of tumor will respond to chemotherapy, and patient will experience long remission. However, upon the completion of chemotherapy, the last two populations of cells persist as residual tumor cells, and they begin to regrow and repopulate the tumor, resulting in recurrence (Figures 1C,D). In this model, quiescent or dormant tumor cells that persist after chemotherapy repopulate the tumor with rapidly proliferating chemotherapy-sensitive tumor cells, thus leading to platinum-sensitive recurrence. This model is supported by the observation of increased density of post-chemotherapy residual tumors having increased cancer stem cells, but recurrences remote from treatment having similar densities of cancer stem cells as the primary tumor (9). With subsequent rounds of platinum-based chemotherapy, the initially small fraction of intrinsically resistant, non-quiescent tumor cells continue to expand, thus leading to eventual platinum resistance. In this model, putative tumor stem cells fit the role of chemotherapy-resistant, quiescent tumor cells that persist after chemotherapy and repopulate the tumor with differentiated, chemotherapy-sensitive tumor cells. Several studies indicate the presence of putative cancer stem cells in ovarian cancer, thus supporting the plausibility of tumor stem cells in platinum-sensitive recurrent ovarian cancer (10, 11).

## Tumor Dormancy as a Mechanism of Platinum-Sensitive Recurrence

In addition to putative cancer stem cells that exist as quiescent, dormant, or intrinsically resistant tumor cells that persist through chemotherapy and repopulate the tumor after chemotherapy, some tumor cells may enter dormancy due to specific tumor microenvironment. It is suggested that cancer cells in transit and cancer cells in unfavorable microenvironment (such as hypoxia, nutrient stress, and lack of growth factors) may enter dormancy (12). For example, Kreso et al. studies indicate that dormant cells in colorectal cancer persist through chemotherapy though they retain potent tumor initiating potential (13). Therefore, after non-quiescent tumor cells are eliminated by chemotherapy and when favorable microenvironment is restored, these dormant cells have the potential to repopulate the tumor (13). In addition, recent studies have shed more light into autophagy as a player in inducing tumor dormancy. Elegant studies by Lu et al. (14), showed that although the tumor suppressor gene *ARHI* promoted autophagy-induced cell death *in vitro*, factors from the tumor microenvironment switched *ARHI*-induced autophagy to a tumor survival mechanism and caused tumor dormancy *in vivo*. Therefore, autophagy and tumor dormancy may constitute another mechanism by which tumor cells persist through chemotherapy and repopulate the tumor upon completion of chemotherapy, thereby resulting in recurrence. Interestingly, Lu et al also show that the inhibition of tumor microenvironment-induced autophagy with the autophagy inhibitor chloroquine results in cell death (14), and therefore autophagy may be therapeutically exploited to minimize tumor dormancy and enhance therapeutic effect of conventional chemotherapy.

## Matrix-Dependent Chemotherapy Resistance as a Possible Mechanism of Platinum-Sensitive Recurrence

Here, we propose an alternate hypothesis for platinum-sensitive recurrence. In this view point, we propose that cancer cells can acquire extracellular matrix (ECM)-dependent platinum resistance (15). These matrix-associated cells persist after chemotherapy and repopulate the tumor after chemotherapy, resulting in recurrence (Figure 1E). Implicit in this hypothetical model is that these cancer cells are not intrinsically resistant to platinum-based therapy. Rather, they are resistant to chemotherapy due to their contact with particular components of ECM. Therefore, tumor repopulated by these persistent residual cells is likely to be sensitive to chemotherapy again if repopulated tumor cells are not in contact with the right components of ECM, thereby resulting in platinum sensitivity. It is important to note that our proposed model represents an alternative hypothesis that seeks to complement and not substitute previous hypothetical models involving tumor stem cells or cancer dormancy. In fact, our proposed hypothetical model may be related to tumor stem cells and cancer dormancy. It is suggested that tumor stem cells exist in particular niche (16, 17) and that specific components of ECM are involved in the establishment of stem cell niche (18). Therefore, it is conceivable that ECM, through its role in the maintenance of stem cell properties, may contribute to chemotherapy resistance. In addition, ECM has been shown to modulate tumor dormancy and serve as a “gatekeeper” in transition from quiescence to proliferation in cancer cells (19). Therefore, it is conceivable that ECM, through its regulation on tumor dormancy, may contribute to chemotherapy resistance.

## Evidence Supporting Matrix-Dependent Chemotherapy Resistance

This proposed model is based on previous studies by various groups indicating that cancer cells grown on specific matrix proteins acquire resistance to chemotherapy (20, 21). For example, Pat Morin and his colleagues have shown that ovarian cancer cells grown on collagen VI are resistant to cisplatin (20). Moreover, cells grown on collagen VI are more resistant than cells grown on collagen III (20), suggesting that acquired resistance is context specific. It is also interesting to note that the initial discovery of the potential role of ECM protein in cisplatin resistance was made from *in vitro* cell line models in which cisplatin-sensitive cell lines were made resistant to cisplatin by exposing the cells to increasing concentrations of cisplatin. Subsequent analysis of gene expression between the cisplatin-sensitive cells and the isogenic cisplatin-resistant cells indicates higher level of collagen VI expression in cancer cells that became resistant to cisplatin (20). These results highlight the dynamic nature of cellular response to cisplatin and suggest that chemotherapy treatment could affect the composition of the ECM by modulating gene expression within cancer cells as well as within stromal cells.

Recent studies that used gene expression profiling technologies also point to a particular group of tumors with pronounced stromal/mesenchymal gene signatures to have worse outcome compared to non-stromal gene signature groups (22, 23). In particular, Helleman et al. suggested that ECM signature is associated with chemotherapy resistance (22). Pathway analysis of gene expression data from tumors with differential response to chemotherapy showed enrichment of ECM signatures in tumors with chemotherapy resistance. In addition, Bowtell and his colleagues showed that two molecular subsets underlies platinum resistance in ovarian cancer: in one subset, cyclin E amplification is associated with platinum resistance, and in another subset without cyclin E amplification, enrichment of cell adhesion, and ECM pathways are associated with platinum resistance (23).

Recently, our own analysis of three datasets [the Cancer Genome Atlas Ovarian Cancer data set (24), Tothill et al. (25), and Bonome et al. (26)] resulted in the identification of several ECM proteins as candidate biomarkers for poor clinical outcomes with respect to recurrence and overall survival (27). In particular, we identified ECM protein fibrillin-1 as a central node in ECM network, and high levels of fibrillin-1 expression in primary tumor are associated with early recurrence in platinum-sensitive ovarian cancer (27). Moreover, another set of gene signature, that is identified from the same study to be associated with early recurrence and early death, consists of nuclear signaling mediated by Fos and Jun nuclear factors (27). These two factors are known to serve as downstream mediators of ECM signaling mediated through integrins (28). Collectively, these two sets of observations point to a potentially significant role of ECM-cell interaction in tumor cell’s response to chemotherapy. It is important to note that expression levels of fibrillin-1 are not associated with platinum-resistant or -refractory ovarian cancer. Rather, it is associated with early recurrence of platinum-sensitive ovarian cancer. Based on these results, we propose a hypothetical model in which ECM, consisting of fibrillin-1 and other components, confers contact-dependent platinum resistance.

In this model, cancer cells not directly attached to specific components of ECM are sensitive to platinum and are eliminated during chemotherapy (Figure 1E). Cancer cells that are directly attached to specific components of ECM (such as Fibrillin-1) are resistant to chemotherapy and persist during chemotherapy. These cells repopulate the tumor giving rise to recurrent ovarian cancer. The amount of residual tumor cells that remain after chemotherapy will be dependent on the amount of ECM, and therefore determine the speed of recurrence. It is conceivable that tumors with a larger component of ECM will have larger amount of residual cells remaining after chemotherapy and quicker recurrence, whereas tumors with smaller component of ECM will have smaller amount of residual cells remaining after chemotherapy and slower recurrence. Results from our immunohistochemical analysis of fibrillin-1 support this view (27). Although ECM components in this model of acquired resistance are tumor extrinsic, it should be noted that the levels of ECM component within tumor microenvironment is a function of tumor intrinsic factors and host intrinsic factors, and therefore intrinsic gene expression within tumor cells may also contribute to differences in ECM deposition and resistance.

## A Unified View

In this unified hypothetical model, in patients with platinum-refractory ovarian cancer, tumor contains intrinsically resistant tumor cells; thus tumor cells are refractory to treatment and progress through treatment (Figure 1A). In patients with platinum-resistant ovarian cancer, i.e., those that recur within 6 months from the last round of chemotherapy, chemo-naïve tumor initially contains heterogeneous populations of chemosensitive as well as intrinsically resistant tumor cells. Upon treatment, chemosensitive tumors were eliminated, thus producing partial treatment response. However, intrinsically resistant tumor cells persist and expand during the treatment, thus leading to early recurrence (Figure 1B). Another scenario might exist whereby these tumors contain a larger component of ECM, which allows a larger component of residual cells to persist after chemotherapy, thereby permitting quicker relapse. If this were true, these tumors that recur within 6 months from the last round of chemotherapy may still contain chemotherapy-sensitive cancer cells and may respond to chemotherapy. In fact, an objective clinical response can be obtained in small percentage of patients with less than 6 months of platinum-free interval, the so called platinum-resistant tumors. Finally, in the last component of the unified hypothesis, patients with platinum-sensitive ovarian cancer are expected to have heterogeneous populations of tumor cells, consisting of putative cancer stem cells, dormant or quiescent tumor cells, and tumor cells that are in contact with specific components of ECM. All these cells are expected to persist after chemotherapy and contribute to platinum-sensitive recurrence (Figure 1F). Eventually, after several rounds of chemotherapy, these cells may evolve to acquire additional genetic alterations leading to acquired resistance. It is also possible that intrinsically resistant tumor cells may exist as a small fraction of total initial tumor bulk. After multiple rounds of chemotherapy, their proportional representation may increase to a point that they eventually dominate the tumor behavior and produce a resistant phenotype.

If proven, the proposed model of matrix-dependent platinum resistance and disease recurrence has several clinical implications. First, although the majority of ovarian cancer cells are intrinsically sensitive to platinum-based chemotherapy, a small fraction of tumor cells acquire matrix-dependent platinum resistance and escape from chemotherapy, leading to recurrence. Second, it will be important to understand the role of ECM components in platinum resistance because enhanced understanding in this area will allow us to design rational therapeutic approaches to eliminate residual cancer cells and provide more durable treatment options. Third, targeting the tumor microenvironment by disrupting cell-matrix interactions may be more “druggable” than targeting putative cancer stem cells because experimental compounds are already available to disrupt cell-matrix interactions (heparin, RGD peptides, integrin inhibitors, etc.) or block kinase signaling initiated by cell-matrix interactions (inhibitors of FAK, Src, PI3K, Akt, etc.). Therefore, small molecule inhibitors and peptides that block upstream ECM signaling or downstream intracellular signaling cascades initiated by ECM signaling should be tested in conjunction with conventional chemotherapy.

Finally, recent studies by Muranen et al. described matrix-dependent resistance to dual-specificity PI3K/mTOR inhibitor BEZ235 and other PI3K or mTOR inhibitors, such as Rapamycin, LY294002, GDC0941, and PIK-90, in ovarian cancer cell lines (29). Therefore, ECM may promote resistance to a broad spectrum of cancer drugs and targeting the ECM and tumor microenvironment may provide significant advances in improving the therapeutic efficacy of conventional as well as emerging novel therapeutics.

## Conclusion

Tumors can be considered as developmental organs defined by abnormal signaling within tumor cells and between tumor cells and their microenvironment. Tumor microenvironment, consisting of (1) cellular components characterized by tumor cells, tumor-associated fibroblasts, immune cells, endothelial cells, and other resident cells, (2) physical components characterized by ECM, and (3) biochemical components characterized by oxygen tension, inflammatory cytokines, chemokines, and growth factors, have long been recognized as a critical determinant of tumor behavior. For example, the activation of v-*src* by Rous sarcoma virus in chick embryo did not produce abnormal growth (30, 31), but when these viral infected tissues were removed from the embryonic microenvironment, they produced a transformed phenotype (32). Similarly, melanoma cells injected into the embryonic microenvironment are reprogramed to remain indolent whereas those cells injected into other microenvironments are capable of inducing abnormal growth (33, 34). Finally, ECM attenuates chemotherapy-induced cytotoxicity in several cancer cell lines from various cancer types (20, 21) – a phenomenon referred to as cell adhesion-mediated drug resistance (35, 36). These studies and others indicate that tumor microenvironment can contribute to tumor dormancy, tumor progression, angiogenesis, metastasis, and chemotherapy resistance (15, 37).

Given the significance of the tumor microenvironment in regulating tumor behavior and, in particular, a tumor cell’s response to chemotherapy, it is important that future drug discovery efforts should include strategies to disrupt cell-matrix interactions or downstream signaling cascades to determine the extent to which these approaches will synergize with conventional chemotherapy to enhance the effectiveness and durability of conventional chemotherapy. Synthetic lethal screens should be performed in more appropriate cellular context, such as 3D culture or matrix-coated cultures to identify drug target genes or drug candidates that synergize conventional chemotherapy.

## Conflict of Interest Statement

The authors declare that the research was conducted in the absence of any commercial or financial relationships that could be construed as a potential conflict of interest.
